# Telehealth mindful exercise for people with knee osteoarthritis: A decentralized feasibility randomized controlled trial

**DOI:** 10.1016/j.ocarto.2024.100494

**Published:** 2024-06-19

**Authors:** Nirali Shah, Natalia Morone, Ehyun Kim, Terry D. Ellis, Ellen Cohn, Michael P. LaValley, Deepak Kumar

**Affiliations:** a Department of Physical Therapy, Boston University, Boston, MA, USA; b Department of Medicine, Boston University Chobanian & Avedisian School of Medicine, Boston, MA, USA; c Department of Occupational Therapy, Boston University, Boston, MA, USA; d Department of Biostatistics, School of Public Health, Boston University, MA, USA

**Keywords:** Digital health, Knee pain, Physical therapy, Psychological impairments, Mindfulness

## Abstract

**Objective:**

Negative psychological beliefs like fear avoidance and catastrophizing can interfere with exercise engagement in people with knee osteoarthritis (OA). Mindfulness, when integrated with exercise, could potentially address both psychological and physical impairments. Our objectives were to optimize and assess the feasibility of a novel telehealth, group-based mindful exercise intervention for people with knee OA.

**Methods:**

We conducted a decentralized randomized controlled trial where participants (n ​= ​40) with symptomatic knee OA were randomized into mindful exercise (n ​= ​21) or exercise-only (n ​= ​19) groups. Both groups received supervised group-based interventions weekly for 8-weeks via Zoom. Primary outcomes were safety, fidelity, and feasibility of the mindful exercise intervention. Participants completed patient-reported outcomes (PRO) for pain, function, and psychological measures at baseline, week-8, and week-14.

**Results:**

Participants were from 21 US states; >90% identified as having White race, 16% were from rural areas, and approximately 40% had an annual income < $50,000. At 8-weeks, mindful exercise and exercise groups had retention rates of 86% (18/21) and 100% (19/19), and attendance was 54% (11.4/21) and 68% (13/19) respectively. There were no adverse events in the mindful exercise group and four in the exercise group related to exacerbation of knee pain. Preliminary findings showed numerically larger improvements in several PROs for the mindful exercise group.

**Conclusion:**

An 8-week telehealth, group-based, mindful exercise intervention was safe for people with knee OA. Our decentralized approach was feasible in terms of recruitment and retention. Further refinement is needed to improve intervention attendance and participant diversity.

## 1 Introduction

About 600 million individuals worldwide have knee osteoarthritis (OA) [1]. People with knee OA typically experience pain with movement, including exercise [2]. When affective responses become coupled with activity or exercise induced pain, negative psychological beliefs like fear avoidance and catastrophizing may develop [3]. Exercise induced pain flares and associated negative psychological beliefs can lead to suboptimal engagement in exercise resulting in high rates of non-response to exercise in people with knee OA [4]. Since inadequate adherence to exercise can spur a cycle of inactivity and further pain and disability, there is a need for interventions that address both physical and psychological impairments related to knee OA.

Mindfulness refers to awareness of thoughts, emotions, and sensations in the present moment without judgement or reactivity [5]. Mindfulness-based meditative practices aim to focus attention on the present experience while disregarding the evaluative or emotional aspects. Hence, mindfulness meditation can help people with chronic pain uncouple affective response from sensations of pain [6]. Mind and body activities like Tai Chi are recommended for people with knee OA [7], but mindfulness has not been applied to exercises typically included in physical therapy (PT) for knee OA. Mindfulness practiced with PT exercises could potentially mitigate the negative psychological beliefs related to the exercises or exercise induced pain and improve exercise adherence.

Implementation of care is often limited by barriers such as travel costs and time, long wait times, and limited availability of specialized services [8]. Telehealth can address these barriers; exercise delivered via telehealth can be as effective as in-person care for people with knee OA [9,10]. Relatedly, decentralized studies, i.e., studies where all procedures are conducted remotely, can be more efficient and inexpensive compared to clinical trials with in-person visits [11].

The National Institutes of Health (NIH) Stage Model is a conceptual framework for developing behavioral interventions that are effective and implementable [12]. Aligned with Stage 1A of NIH Stage Model, our objective was to optimize a telehealth group-based mindful exercise intervention for people with knee OA using a decentralized randomized controlled trial (RCT) design. The results from this study will guide further refinement of the mindful exercise intervention (Stage 1B) prior to larger efficacy trials (Stage 2).

## 2 Methods

### 2.1 Design

We conducted a decentralized, single-site, parallel-arm, feasibility RCT with 1:1 allocation. Recruitment, intervention delivery, and outcome assessment were all conducted remotely. The trial protocol was prospectively registered and can be accessed at ClinicalTrials.gov (NCT05524116). Findings of the trial are reported in accordance to the Consolidated Standards of Reporting Trials (CONSORT) extension for pilot or feasibility studies [13]. The study was approved by Boston University's Institutional Review Board (protocol #6492). All participants provided informed consent electronically before participating in the study.

### 2.2 Participants

Recruitment occurred from October 2022 to March 2023 primarily through social media. Supplemental recruitment strategies included Craigslist website, local senior and community centers, and existing database of participants from prior studies. Recruitment was initially limited to the Boston area and was eventually expanded to the contiguous United States. Inclusion criteria were age ≥50 years, presence of activity related pain, morning stiffness ≤30 ​min, body mass index <40 ​kg/m^2^, knee pain on most days for ≥3 months, pain severity ≥4/10 over previous week, access to computer and internet for telehealth sessions, ability to speak and understand English, and available for study duration. Exclusion criteria were contra-indications to exercise, more pain in lower back or legs than knee, other treatments for knee (i.e., physical therapy, mindfulness, injections, surgery) in previous six months, planning for OA treatment during study period, history of diseases (musculoskeletal, neurological, cancer) that involve the knee, pregnancy, participation in another trial for musculoskeletal pain, and suspected or known drugs or alcohol use disorder. Interested individuals completed an online screening form and those who qualified participated in additional screening over phone. If eligible, participants signed an electronic informed consent [14], and were enrolled in the study. Enrollment occurred in two waves; interventions were initiated for each wave once enough participants were enrolled to form groups. A sample size of 40 was calculated based on an expected attendance of 80% to telehealth sessions with a confidence level of 95% and a margin of error of ±12%.

### 2.3 Randomization, blinding, and telehealth training

The randomization list was computer-generated, using permuted blocks stratified by sex, by study biostatistician (MPL) who did not have any interaction with the participants. After enrollment and baseline assessments, participants completed randomization and telehealth training via HIPAA-protected Zoom. During this visit, a researcher (NS) who was unaware of the assignment prior to the visit, opened sealed sequentially numbered envelopes and informed participants of their group assignment. Participants were informed that both groups were receiving active interventions, and they were blinded to the study hypotheses [15]. After group assignment, the researcher provided training on how to use Zoom for the interventions, guidance on setting up home space for interventions, and gave detailed information about their intervention to participants using a program manual.

### 2.4 Interventions

Details on interventions are provided in accordance with the TIDieR checklist [16]. The interventions for both groups were for 8-weeks, delivered weekly in a supervised group setting (n ​= ​6 in wave 1, n ​= ​15 in wave 2 for mindful exercise; n ​= ​8 in wave 1, n ​= ​11 in wave 2 for exercise) via HIPAA-protected Zoom. Participants in both groups were given exercise equipment (resistance bands, step-up platform with adjustable height) and weekly OA-education materials via email. The participants were also provided with a program manual with information about the intervention, links to online videos of exercises, and audio recordings of guided meditations (only for mindful exercise group). The educational materials were developed by the study team and included topics such as: knee OA overview, pain education, comorbidities in knee OA, physical activity and exercise, weight management, reducing joint loading, complementary and alternative therapies, and pharmacological and surgical therapies. After the 8-week interventions, participants were asked to continue to practice on their own for another 6 weeks.

#### 2.4.1 Exercise group

The exercise program was delivered by a physical therapist (NS). Each 1-h session included warm-up, 5–6 lower extremity strengthening exercises, 1–2 neuromuscular and balance exercises, and cool-down (Table 1). Before the start of each set, the interventionist demonstrated the exercise. Each exercise was performed bilaterally in three sets of 8–12 repetitions at an intensity level corresponding to a Rating of Perceived Exertion (RPE) of 4–6 on the modified Borg CR10 scale [17]. Participants were suggested to progress to a more challenging exercise if their RPE was <4 while performing the exercise with proper technique. For home exercises, participants were instructed to perform strengthening exercises at least two other days a week and aerobic exercise of their choice at least five days a week.

**Table 1:** List of supervised exercises and meditations.

Goals	Exercises: *Each session included 6–8 of the following exercises.* *All exercises were done bilaterally in 3 sets of 8 repetitions*
**Strengthening**	

*Knee Extensors*	Seated knee extension
	Standing terminal knee extension
*Knee Flexors*	Leg Curls
*Hip Extensors*	Backward leg raises
*Hip Flexors*	Forward leg raises
*Hip Abductors*	Side leg raises
*Hip Adductors*	Standing hip adduction
*Calf muscles*	Heel raises
**Neuromuscular**	Step ups
	Squats
	Lunges

**Goals**	**Meditations**
**Session 1**	
*Raisin meditation*	Training on how to separate senses of observation, touch, sound, and feel
*Awareness of breath meditation*	Training on how to use breath as an anchor
**Session 2**	
*Body scan meditation*	Training on how to use body sensation as an anchor
*Breath and counting meditation*	Training on how to use counting as an anchor
**Session 3**	
*Moving meditation*	Introducing how to combine movement and mindfulness
*Body scan meditation*	Practice on how to use body sensation as an anchor
*Mindful exercises*	Combining exercise and mindfulness
**Session 4**	
*Breath and counting meditation*	Training on how to exercise using either the breath, counting, or body sensation as an anchor
*Walking meditation*	Combining movement and mindfulness
*Mindful exercises*	Combining exercise and mindfulness
**Session 5**	
*Awareness of breath meditation*	Practice on how to use breath as an anchor
*Mindful exercises*	Combining exercise and mindfulness
**Session 6**	
*Awareness of breath meditation*	Practice on how to use breath as an anchor
*Body scan meditation*	Practice on how to use body sensation as an anchor
*Mindful exercises*	Combining exercise and mindfulness
**Session 7**	
*S.T.O.P meditation (S=sit down, T=take a breath, O=observe, P=proceed with day)*	Training on incorporating mindfulness in daily life
*Pain-focused meditation*	Training on how to detach from difficult sensations
*Mindful exercises*	Combining exercise and mindfulness
**Session 8**	
*Review and summary*	A summary and practice of what was covered in the 8-week program and practice of aware of breath and body scan meditations
*Mindful exercises*	Combining exercise and mindfulness

#### 2.4.2 Mindful exercise group

The mindful exercise intervention was delivered jointly by the physical therapist (NS) and a mindfulness instructor with ∼20 years of experience. The mindfulness components of the intervention were adapted from Mindfulness-Based Stress Reduction (MBSR) program and investigator's (NM) prior work with mindfulness for chronic low back pain [5,18]. The curriculum included a review of biopsychosocial model of pain processing, its relationship to mindfulness, and chair and standing meditations focused on breath, body scan, or counting (Table 1). The innovative components of the program included integration of mindfulness with exercise. Each session was ∼2 ​h in length divided into four 30-min blocks. In first and third 30-min blocks, participants were trained to develop moment-to-moment awareness using breath, body sensations, or counting as an anchor. In the second and the fourth 30-min blocks, participants performed the same exercises as the exercise group. In the first two sessions, the mindfulness and exercise components were kept separate. Starting with session 3, participants were instructed to practice mindfulness (i.e., use breath, body sensation, or counting as an anchor) while performing exercise movements. For home practice, participants were advised to practice strengthening exercises mindfully at least two days each week and any aerobic exercise mindfully at least five days each week. They were also asked to practice meditations for at least 20-min each day.

### 2.5 Safety, fidelity, and feasibility

We assessed safety as number of adverse events (AEs) and serious adverse events (SAEs) related to the study. AE was defined as any unfavorable or unintended diagnosis, sign (including abnormal laboratory finding), symptom, or diseases temporarily observed during the study intervention. AEs included any new events that were not present during the pre-intervention period or events that were present during the pre-intervention period but increased in severity. SAE was defined as any AE that results in any of the following: death, life-threatening adverse experience, inpatient hospitalization or prolongation of existing hospitalization, persistent or significant disability/incapacity, congenital anomaly/birth defect, or cancer, or any other experience that suggests a significant hazard, contraindication, side effect or precaution that may require medical or surgical intervention to prevent one of the outcomes listed above, or event that changes the risk/benefit ratio of the study.

We assessed fidelity as home adherence to intervention (self-reported weekly via electronic surveys [19] and participants’ feedback about the interventionist and sessions using custom surveys administered post-intervention. We assessed feasibility as recruitment, attendance, and retention. We defined, *a priori,* excellent, and good benchmarks for each outcome. Excellent and good recruitment was defined as ≥ 40 or ≥30 participants enrolled over 6-months, respectively. Excellent and good retention was defined as ≥ 80% and ≥60% completion of post-intervention assessments, respectively. Excellent and good attendance was defined as ≥80% and ≥60% average attendance during online sessions, respectively.

### 2.6 Other outcomes

Participants electronically completed several patient-reported outcomes (PROs) that were selected to reflect outcomes recommended for clinical trials of interventions for knee OA including as primary (i.e., OA related pain, function) and secondary (i.e., medication use, global rating, movement-evoked pain, pain interference, widespread pain) outcomes [20], as well as potential mediators of intervention effects (i.e., adherence, mindfulness, catastrophizing, fear avoidance, self-efficacy, mood) [19]. We defined the index knee as the more painful knee, or a knee selected at random if the pain was similar in both knees. We designated the 8-week timepoint as the primary for PROs.

At baseline, all participants self-reported demographic data including sex, race, education, family income, tobacco use, and employment status from a fixed set of categories. Outcomes assessed at baseline (before randomization), post-intervention (week 8), and at follow-up (week 14) included OA related pain, function, symptoms, quality of life (Knee Injury and Osteoarthritis Outcome Score, KOOS Pain, range 0–100) [21], pain interference during daily activities (Pain, Enjoyment, and General Activity scale, PEG, range 0–10) [22], mindfulness (Cognitive and Affective Mindfulness Scale Revised, CAMS-R, range 12–48) [23], mood (8-item Patient Health Questionnaire, PHQ-8, range 0–24) [24], and global rating of OA (Patient Global Assessment of Osteoarthritis, PGA-OA, range 0–100) [25].

Additionally, participants completed the following measures at baseline and post intervention (week-8): catastrophizing (3-item Pain Catastrophizing Scale, PCS-3, range 0–12) [26], fear avoidance (Fear Avoidance Beliefs Questionnaire for Physical Activity, FABQ-OA, modified for knee OA, range 0–24) [27], count of painful joints for widespread pain [28], neuropathic pain (PainDETECT, range 0–38) [29], OA-related self-efficacy (Arthritis Self-Efficacy Scale, ASES, range 1–10) [30], and chronic pain related self-efficacy (Chronic Pain Self-Efficacy Scale, CPSS, range 0–100) [31]. Outcomes assessed each week during the intervention and follow-up periods included movement-evoked pain (numeric rating for pain during nominated most painful activity, NRS-na, range 0–10) [32] and oral analgesic use for knee pain. Finally, feedback, and satisfaction surveys were assessed only at week-8, and Patient Global Impression of Change (PGIC) [33] was assessed at week-8 and week-14.

### 2.7 Statistical analyses

We descriptively report number of adverse events, and data for recruitment, attendance, and retention. We report aggregate data for intervention adherence at home and feedback from participants. For other PROs, we report within group changes and differences between groups along with 95% confidence intervals. Data are reported using an intention-to-treat approach. Missing data were not imputed.

## 3 Results

**Fig. 1:**
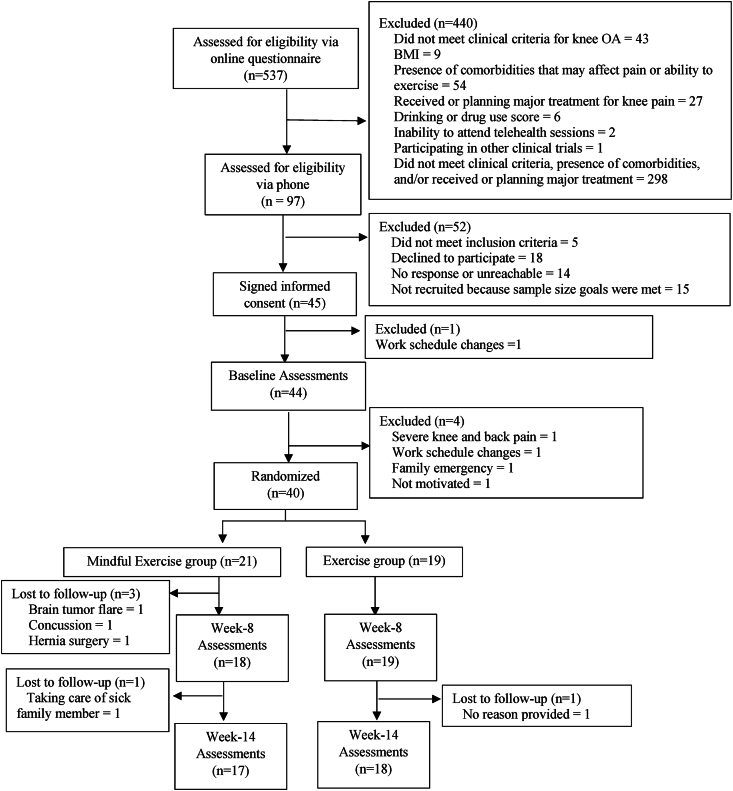
CONSORT diagram.

Over 6-months, 537 people were screened online for eligibility, 97 (18 %) were eligible for phone screening, 45 (∼8%) completed baseline assessments, and 40 (∼7%) were enrolled and randomized into mindful exercise (n ​= ​21) or exercise group (n ​= ​19) (Fig. 1). Enrolled participants were from 21 US states; 7 (15.6%) resided in rural areas [34], >90% self-identified as White, and ∼40% reported annual income of <$50,000 (Table 2).

**Table 2:** Participant Characteristics. *Data reported as number (%) unless specified.*

	Mindful Exercise (n ​= ​21)	Exercise (n ​= ​19)
**AGE, mean (SD)**	63.8 (8.9)	67.4 (7.4)
**SEX**		
*Female*	18 (85.7)	17 (89.5)
*Male*	3 (14.3)	2 (10.5)
**BMI, mean (SD)**	31.5 (4.4)	26.7 (4.9)
**RACE**		
*White*	19 (90.5)	18 (94.7)
*Black (incl. African American) or Asian or not reported*^a^	2 (9.5)	1 (5.3)
**EDUCATION**		
*High school or College 1 – 3 years*^a^	8 (38.1)	6 (31.6)
*Undergraduate, graduate, or doctorate*^a^	13 (61.9)	13 (68.4)
*Did not report*		
**INCOME**		
*Less than $50,0000*^a^	9 (42.9)	8 (42.1)
*More than $50,000*^a^	11 (52.4)	10 (52.6)
*Did not report*	1 (4.8)	1 (5.3)
**EMPLOYED**	9 (42.9)	6 (31.6)
**MARITAL STATUS**		
*Married*	10 (47.6)	9 (47.4)
*Widowed, divorced, single, or not reported*^a^	11 (52.4)	10 (52.6)

a Categories combined to protect privacy.

### 3.1 Safety and fidelity

There were no SAEs in either group. There were no AEs related to the study in mindful exercise group. There were four reports of exacerbation of knee pain in exercise group. Over 8-weeks, mindful exercise group engaged in strengthening exercises on average 4 days/week and exercise group on average 3 days/week (Table 3). Both groups engaged in aerobic exercises 5 days/week during the intervention period and sustained the frequency of strengthening and aerobic exercises at follow-up. Total exercise time exceeded 150 ​min/week for both groups, with exercise group reporting more exercise time (∼300 vs 220 ​min/week). Participants in mindful exercise group practiced mindfulness ∼5 days/week during intervention and follow-up periods.

**Table 3:** Self-reported at-home adherence. *Data reported as mean (95% confidence intervals)*.

	Mindful Exercise Group^a^			Exercise Group^a^		
	Overall	Over 8 weeks	Last 6 weeks	Overall	Over 8 weeks	Last 6 weeks
**Strengthening Exercises**						
*Total Minutes per week*	57.5 (52.4, 62.6)	61.9 (55.7, 66.8)	48.6 (39.8, 57.4)	72.9 (60.8, 85.0)	80.6 (64.0, 97.2)	61.7 (44.2, 79.2)
*Days per week*	3.9 (3.5, 4.2)	3.8 (3.3, 4.2)	4.1 (3.3, 4.8)	2.6 (2.3, 3.0)	2.7 (2.3, 3.2)	2.5 (2.0, 3.0)
**Aerobic Exercises**						
*Total Minutes per week*	164.4 (139.4, 189.5)	165.2 (139.6, 190.8)	162.9 (106.2, 219.5)	232.3 (205.5, 259.0)	230.8 (195.8, 265.8)	234.4 (192.1, 276.8)
*Days per week*	4.9 (4.6, 5.3)	4.7 (4.3, 5.2)	5.3 (4.6, 6.1)	4.9 (4.7, 5.2)	4.9 (4.6, 5.3)	5.0 (4.5, 5.4)
**Mindfulness Practice**						
*Total Minutes per week*	93.3 (82.4, 104.2)	93.7 (83.5, 103.8)	92.6 (66.0, 119.1)			
*Days per week*	5.1 (4.7, 5.4)	4.9 (4.5, 5.3)	5.5 (4.9, 6.2)			
**Oral Analgesic Use**						
*% of participants who took oral analgesics*	33.1 (25.7, 40.6)	38.1 (28.7, 47.5)	23.1 (11.2, 34.9)	50.2 (43.3, 57.2)	47.1 (38.0, 56.2)	54.9 (43.9, 65.9)
*Days per week of oral analgesic use*	1.3 (0.9, 1.6)	1.6 (1.1, 2.1)	0.7 (0.3, 1.0)	2.0 (1.7, 2.3)	1.8 (1.4, 2.3)	2.3 (1.7, 2.8)

a Number of participants who provided weekly data varied by group and week.

Oral analgesic use for knee pain was on average 2 days/week in both groups over 8-weeks (Table 3). Over 6-week follow-up, this was reduced to 1 day/week in mindful exercise group but not in exercise group. The proportion of oral analgesic use for knee pain was lower in mindful exercise group during intervention period (38% vs. 47%) which was further magnified during 6-week follow-up (23% vs. 55%).

**Fig. 2:**
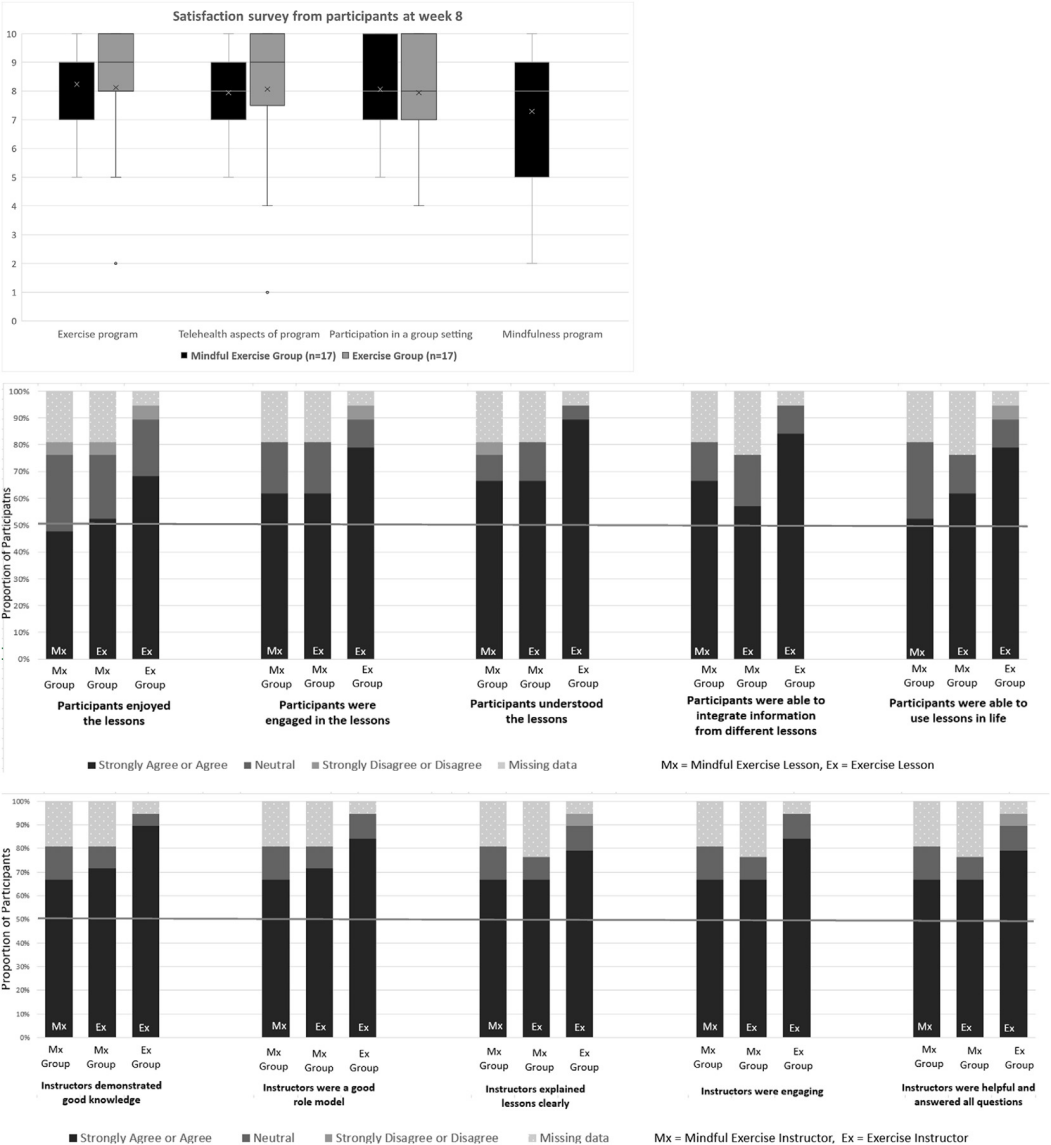
(TOP) Satisfaction data from participants at week 8 on an 11-point numeric rating scale ranging from Not Satisfied (0) to Extremely Satisfied (10). (MIDDLE) Proportion of participants in response categories of feedback items about intervention sessions. (BOTTOM) Proportion of participants in response categories of feedback items about instructors.

Satisfaction ratings were on average ≥7/10 for exercise program, mindfulness program, telehealth, and group aspects of the intervention (Fig. 2). Majority of participants agreed or strongly agreed with positive attributes of the mindfulness and exercise instructors (67–89%) (Fig. 2). Many of the participants in the exercise group (68–89%) provided positive feedback about the intervention**.** However, somewhat smaller proportion (48–78%) provided positive feedback about mindful exercise intervention. Additional context for these ratings was available via open-ended questions in the survey (Table 4). The mindful exercise group expressed desire for shorter frequent sessions, and reported challenges with selected exercises, exercise equipment, and with learning mindfulness. The preferences related to exercise were echoed by the exercise group. Additionally, the exercise group expressed desire for individual guidance and challenges in coordinating exercises with larger group.

**Table 4:** Aspects of the intervention that participants were satisfied and not satisfied with.

	Mindful Exercise Group (n ​= ​17)		Exercise Group (n ​= ​18)	
	Satisfied	Not Satisfied	Satisfied	Not Satisfied
Exercises	• Variety of exercises • Provisions for exercise modifications according to one's abilities • Exercise equipment (i.e., resistance bands and step riser) • Real time feedback and guidance • Repeatability of exercises to help develop a routine • Visual demonstration of exercises • Enjoyed learning new exercises	• Found the intensity of the exercises aggravating on the knee, despite modifications for adaptation • Desire for more instruction on how to use exercise equipment • Frustration and challenges with exercise equipment (band, step up)	• Specific strengthening exercises for the knee • Perceived improvements in functional abilities • • Increased confidence in ability because of exercising at an intensity higher than previously engaged in Email summarizing the exercises • Real time feedback and guidance • Consistency and regularity of exercises • Visual demonstration of exercises • Enjoyed learning new exercises	• Desire for more individualized exercise plan • Exercises felt hurried and not enough demonstration of exercises • Found the exercises challenging • Desire for more guidance on exercises, especially home exercises • Afraid of falling on using exercise equipment • Frustration and challenges with exercise equipment (band, step up)
Mindfulness	• Enjoyed meditations • Ability to learn a new way of thinking and reactions • Helped focus on different body parts to reduce discomfort • Relaxation, concentration, and inner peace that mindfulness provided	• • Preference for silence after instructions on the meditation instead of guided meditations throughout Found mindfulness difficult to learn and incorporate	N/A	N/A
Telehealth setting	• Ability to do intervention in own space	• Can only look at instructor from one angle • Challenges with hearing or seeing the instructor • Did not like seeing self on the screen	• Time-saving • Not having to travel or find parking in Boston • Convenient	• Challenges with some zoom features (muting, raising volume, using chat feature, etc.) • Challenges with hearing or seeing the instructor • Challenges with access to the right technology (laptop was perceived as better compared to iPad)
Group-setting	• Enjoyed group setting • Liked discussion for questions that one would not have thought of or didn't want to ask	• Desire for peer interaction	• Motivating to be part of a group • Fun • Liked meeting other people • Liked not exercising alone	• Felt challenging to co-ordinate exercises with everyone in the group • Desire for peer interaction to discuss experiences in managing pain and exercises • Preference for individual one-on-one physical therapy
Instructors and supervision	• Enjoyed pace of instruction • Access to instructors for questions or comments • Confidence in instructor's abilities • Instructors were knowledgeable, engaging, and had clear communication		• Instructor was knowledgeable, supportive, encouraging, challenging, and attentive	• Unable to understand the instructor
Other	• Self-explanatory program manual that enabled a self-directed program at home • Pain education content • Access to instructors for questions or comments	• Preference for shorter (1 ​h) but frequent sessions (e.g., twice a week) • Preference for a different time for the session • Desire for video recording of the sessions	• Enjoyed education content and material	• Challenges with doing the exercises on own • Desire for video recording of the sessions

### 3.2 Feasibility and other outcomes

At week-8, 86% (18/21) in mindful exercise group and 100% (19/19) in exercise group, and at week-14, 81% (17/21) in mindful exercise group and 95% (18/19) in exercise group provided partial or complete PRO data. On average, participants attended 54% and 68% of the sessions in mindful exercise and exercise groups, respectively. Four participants in the mindful exercise group and one in the exercise group did not attend any sessions due to reasons unrelated to the study (Fig. 1). Among those who attended at least one session, average attendance was 67% and 72% of the sessions in mindful exercise and exercise groups, respectively. In the mindful exercise group, 62% (13/21), 57% (12/21), and 43% (9/21) of the participants attended at least 50%, 60%, and 80% of the sessions while 79% (15/19), 74% (14/19), and 58% (11/19) of the participants in the exercise group met these thresholds.

Results from the PROs are shown in Table 5, Table 6. From baseline to week-8, we observed numerically larger improvements in KOOS Pain, ADL, S/R, and QOL subscales, NRS-na, PGA-OA, PEG, and PCS in mindful exercise vs exercise group. However, improvements in KOOS Symptoms were numerically greater for exercise group. The differences between groups were further magnified from baseline to week-14 in favor of mindful exercise group for KOOS Pain, ADL, S/R, and QOL subscales, PEG, NRS-na, and PGA-OA. Participant's global impression of change was similar in both groups at week-8 and week-14.

**Table 5:** ePROs. Data reported as mean (95% confidence interval), unless otherwise mentioned.

ePRO (range)	Mindful Exercise Group			Exercise Group		
	Week 0 (n ​= ​21) b	Week 8 (n ​= ​17) b	Week 14 (n ​= ​17)	Week 0 (n ​= ​19) b	Week 8 (n ​= ​18) b	Week 14 (n ​= ​18) b
**KOOS (0**–**100)**						
*Pain*	57.7 (52.6, 62.7)	61.8 (54.4, 69.1)	64.2 (57, 71.4)	61.3 (57.6, 64.9)	63.7 (57.9, 69.5)	63.8 (57.3, 70.3)
*Symptoms*	59.4 (53.9, 65)	59.9 (53.7, 66.1)	62.1 (54, 70.3)	59.7 (54.3, 65)	63.1 (58.5, 67.8)	68.8 (63, 74.5)
*ADL*	63 (57.8, 68.1)	68.2 (60.3, 76.1)	68.5 (62.4, 74.6)	72.1 (66.7, 77.6)	72.6 (66.4, 78.8)	71.4 (64.2, 78.7)
*Sports and Recreation*	37.4 (31.1, 43.7)	40.6 (32.4, 48.7)	45.3 (36.5, 54.1)	48.1 (37.5, 58.6)	47.2 (34.2, 60.3)	42.8 (30.6, 55.1)
*QOL*	41.1 (35.1, 47.1)	48.5 (42.4, 54.6)	52.6 (44.4, 60.7)	45.8 (41.6, 50)	49 (44.1, 53.8)	52.1 (46.4, 57.8)
**NRSna (0–10)**	6.5 (6.1, 6.9)	5.2 (4.4, 5.9)	4.8 (3.8, 5.7)	6.1 (5.3, 6.9)	5.1 (4, 6.2)	4.8 (3.8, 5.9)
**PHQ-8 (0**–**24)**	3 (1.7, 4.3)	3.9 (1.9, 6)	4.1 (1.9, 6.3)	4.6 (2.3, 6.9)	3.7 (2.2, 5.3)	3.9 (2.4, 5.5)
**CAMS-r (12**–**48)**	37.4 (34.5, 40.3)	36.4 (33, 39.9)	37.3 (33.4, 41.2)	35.3 (32.7, 37.8)	33.9 (30.6, 37.3)	35.2 (32.1, 38.4)
**PGA-OA (0**–**100)**	63.7 (54.7, 72.6)	65.7 (56.9, 74.5)	68.7 (60.7, 76.7)	59.8 (49.2, 70.4)	64.5 (54.5, 74.5)	60.2 (50.8, 69.5)
**PEG (0–10)**	5.4 (4.6, 6.1)	3.9 (3, 4.7)	3.4 (2.5, 4.4)	4.9 (4.2, 5.6)	4 (2.9, 5)	3.9 (2.9, 4.9)
**PainDETECT (0–38)**	8.5 (6.6, 10.4)	7.6 (4.9, 10.4)	–	9.8 (7.1, 12.4)	9.4 (7, 11.8)	–
**PCS (0–12)**	3.9 (2.9, 4.9)	3.1 (2, 4.1)	–	3.5 (2.7, 4.4)	3.4 (2.3, 4.6)	–
**FABQ-PA (0–24)**	15.5 (12.9, 18.1)	14.8 (11.6, 18.1)	–	18.3 (16.1, 20.5)	16.7 (13.4, 20)	–
**Widespread Pain Index (0–12)**	3.1 (2.2, 4.1)	3.7 (2.6, 4.9)	–	3.9 (3, 4.8)	4.1 (2.9, 5.3)	–
**ASES (1–10)**						
*Pain*	6.1 (5.6, 6.6)	6.2 (5.3, 7.1)	–	6.5 (5.8, 7.3)	7.1 (6.3, 7.9)	–
*Function*	7.7 (7.1, 8.2)	8 (7.2, 8.8)	–	8 (7.4, 8.7)	8.1 (7.2, 9.1)	–
*Other Symptoms*	6.8 (6.2, 7.3)	7.2 (6.4, 8.1)	–	7 (6.2, 7.7)	7.1 (6.3, 7.9)	–
**CPSS (0**–**100)**						
*Pain*	66.2 (59.6, 72.7)	66.9 (59.1, 74.8)	–	71.5 (64.6, 78.4)	66.8 (56.8, 76.8)	–
*Function*	81.4 (74.7, 88.1)	78.2 (66.5, 89.9)	–	86 (78.8, 93.2)	82.2 (72.3, 92.2)	–
*Coping*	72.6 (66.3, 78.8)	70.5 (60.6, 80.5)	–	68.1 (60.1, 76)	71.2 (62.1, 80.3)	–
**PGIC (-7, 7)**^a^	–	2.4 (1.2, 3.7)	2.2 (1.1, 3.3)	–	2.2 (1, 3.4)	1.4 (0.1, 2.7)

At baseline, mindful exercise group n ​= ​21 except for PainDETECT (n ​= ​20) and CPSS coping subscale (n ​= ​20).

At baseline, exercise group n ​= ​19 except for PainDETECT (n ​= ​18), Widespread pain index (n ​= ​18), CPSS function subscale (n ​= ​18), and CPSS coping subscale (n ​= ​18).

At week-8, mindful exercise group n ​= ​17 except for PainDETECT (n ​= ​16), ASES other symptoms subscale (n ​= ​16), CPSS function subscale (n ​= ​16), CPSS coping subscale (n ​= ​16), and PGIC (n ​= ​16).

At week-8, exercise group n ​= ​18 except for PHQ-8 (n ​= ​19), ASES all subscales (n ​= ​19), CPSS pain (n ​= ​17), and CPSS function (n ​= ​16).

At week-14, exercise group n ​= ​18, except for CAMS-r (n ​= ​17).

PEG = Pain, Enjoyment, and General Activity scale; NRSna ​= ​Numeric Rating Scale in nominated activity; KOOS = Knee Injury and Osteoarthritis Outcome Score; ASES = Arthritis Self-efficacy Scale; PHQ-8 ​= ​Patient Health Questionnaire; PCS = Pain Catastrophizing Scale; WPI = Widespread Pain Index; CAMS-r ​= ​Cognitive Affective Mindfulness Scale; PGA-OA = Patient Global Assessment of Osteoarthritis; CPSS = Chronic Pain Self-efficacy Scale; PGIC = Patient Global Impression of Change.

a Median (range).

b Week 8 and Week 14 data includes participants who fully completed ePRO measures.

**Table 6:** Within group and between group difference in patient reported outcomes. *Data reported as mean (95% confidence interval)*.

	Mindful Exercise Group (n ​= ​17)	Exercise Group (n ​= ​18)		Mindful Exercise Group (n ​= ​17)	Exercise Group (n ​= ​18)	
	Within group difference (baseline to week 8)^a^		Between group difference (baseline to week 8)	Within group difference (baseline to week 14)^a^		Between group difference (baseline to week 14)
**KOOS (0**–**100)**						
*Pain*	5.7 (−2.1, 13.6)	2.1 (−1.8, 6.1)	−3.6 (−13.1, 5.9)	**8.2 (0.7, 15.7)**	2.2 (−2.6, 7)	−6.0 (−15.5, 3.6)
*Symptoms*	0.8 (−5.7, 7.2)	2.5 (−3.2, 8.3)	1.8 (−7.4, 11)	3.0 (−5.7, 11.7)	**8.2 (0.3, 16.7)**	5.2 (−7.3, 17.7)
*ADL*	7.3 (0.4, 14.1)	−0.2 (−4, 3.5)	−7.5 (−15.9, 0.9)	7.6 (1, 14.2)	−1.4 (−5.5, 2.7)	−9.0 (−17.4, −0.7)
*Sports and Recreation*	3.8 (−4.2, 11.8)	−3.2 (−9.9, 3.4)	−7.1 (−18.2, 4)	**8.5 (-0.4, 17.5)**	−6.7 (−20.2, 6.8)	−15.2 (−32.6, 2.2)
*QOL*	**8.1 (0.5, 15.7)**	2.7 (−1.8, 7.2)	−5.4 (−14.9, 4.1)	**12.1 (2, 22.3)**	5.8 (0.7, 10.9)	−6.3 (−18.6, 6)
**NRSna (0–10)**	**−1.4 (-2.3, -0.5)**	**−1 (-1.8, -0.2)**	0.4 (−0.9, 1.7)	**−1.8 (-2.8, -0.8)**	**−1.3 (-2.1, -0.5)**	−0.5 (−0.8, 1.9)
**PHQ-8 (0**–**24)**	0.9 (−0.5, 2.3)	−0.9 (−2.5, 0.7)	−1.8 (−4.1, 0.4)	1.1 (−0.5, 2.7)	−0.7 (−2, 0.7)	−1.8 (−4, 0.4)
**CAMS-r (12**–**48)**	−0.8 (−3.6, 2.1)	−0.9 (−3, 1.1)	−0.2 (−4, 3.6)	0.1 (−3.5, 3.8)	0.8 (−1.7, 3.4)	0.7 (−4.1, 5.5)
**PGA-OA (0**–**100)**	4.8 (−7.6, 17.2)	1.4 (−5.4, 8.2)	−3.4 (−18.7, 11.8)	7.8 (−4, 19.6)	0.5 (−12.9, 14)	−7.3 (−26.5, 11.9)
**PEG (0–10)**	**−1.7 (-2.7, -0.7)**	**−1 (-1.8, -0.2)**	0.7 (−0.6, 2.1)	**−2.2 (-3.3, -1.1)**	**−1.1 (-1.7, -0.4)**	1.1 (−0.2, 2.5)
**PainDETECT (0–38)**	−0.9 (−3.2, 1.4)	−0.1 (−2.7, 2.4)	0.8 (−2.9, 4.4)			
**PCS (0–12)**	−1.2 (−2.3, −0.2)	0 (−0.7, 0.7)	1.2 (−0.1, 2.6)			
**FABQ-PA (0–24)**	0.4 (−2.4, 3.2)	−1.6 (−4.1, 0.8)	−2 (−6, 1.9)			
**WPI (0–12)**	0.7 (−0.1, 1.5)	0.4 (−0.5, 1.2)	−0.3 (−1.6, 0.9)			
**ASES (1–10)**						
*Pain*	0 (−1, 1)	0.6 (−0.1, 1.3)	0.6 (−0.7, 1.9)			
*Function*	0.3 (−0.2, 0.9)	0.1 (−0.5, 0.8)	−0.2 (−1.1, 0.7)			
*Other symptoms*	0.5 (−0.6, 1.6)	0.1 (−0.4, 0.6)	−0.4 (−1.7, 0.9)			
**CPSS (0**–**100)**						
*Pain*	−1.5 (−9.7, 6.7)	−3.6 (−8.5, 1.2)	−2.1 (−12.3, 8.1)			
*Function*	−4 (−14.2, 6.1)	−3.5 (−9, 2.1)	0.5 (−12, 13.1)			
*Coping*	−5.6 (−15.5, 4.3)	2.5 (−1.9, 6.9)	8.1 (−3.8, 19.9)			

ASES = Arthritis Self-efficacy Scale; CAMS-r ​= ​Cognitive Affective Mindfulness Scale revised; CPSS = Chronic Pain Self-efficacy Scale; FABQ = Fear Avoidance Beliefs Questionnaires modified for knee OA; KOOS = Knee Injury and Osteoarthritis Outcome Score; NRSna ​= ​Numeric Rating Scale in nominated activity; PCS ​= ​3-item Pain Catastrophizing Scale; PEG = Pain, Enjoyment, and General Activity scale PGA-OA = Patient Global Assessment of Osteoarthritis; PHQ-8 ​= ​8-item Patient Health Questionnaire; WPI = Widespread Pain Index.

For between group difference, greater within group change for mindful exercise vs exercise group denoted as negative values for ASES, CAMS-r, CPSS, KOOS, and PGA-OA, and positive values for FABQ-PA, NRS, PainDETECT, PEG, PHQ-8, PCS, and WPI.

Bolded numbers represent improvements that are ≥ the minimal clinically important difference for the scale; KOOS QOL ​= ​8 points [21], NRS ​= ​1 point [48], and PEG scale ​= ​0.6–1.22 [49].

a For within group change, improvements noted as positive values for ASES, CAMS-r, CPSS KOOS, and PGA-OA, and negative values for FABQ-PA, NRS, PainDETECT, PCS, PEG, PHQ-8, and WPI.

## 4 Discussion

Our goal was to optimize a novel telehealth group-based mindful exercise intervention for people with knee OA aligned with Stage 1A of the NIH Stage Model. Overall, the mindful exercise intervention was safe and satisfactory. Our decentralized approach was feasible for recruitment and retention. Our findings indicate the need for further refinement of mindful exercise intervention to promote greater adherence and modifications to decentralized strategies to improve racial diversity of the cohort.

There were no adverse events in the mindful exercise group (vs. 4 in exercise group). Although we were not powered to compare analgesic use between groups, our preliminary results suggest that a smaller proportion of participants in mindful exercise group used oral analgesics for their knee pain. Importantly, the use of oral analgesics appears to be reduced during and after the intervention. These preliminary findings suggest that exercise may be more tolerable when combined with mindfulness and provide the necessary premise for further investigation of this approach. Notably, the greater volume of exercise was reported in the exercise group and these findings need to be confirmed in larger studies. The total time spent engaging in exercise on average each week was greater than 150 ​min/week for both groups meeting the public health guidelines [35]. Also, participants in both groups adhered to our prescribed dosage of exercise (i.e., at least 2 days of strengthening exercise in addition to the intervention session and at least 5 days of aerobic activity each week). Participants also reported performing meditation on average 5 days/week as we had prescribed, suggesting a willingness to engage in mindfulness methods outside of the formal exercise sessions.

Feedback for both groups showed that in general, participants enjoyed the lessons, were engaged, and were able to use the skills in their daily lives. Similarly, participants were generally satisfied with the intervention content, as well as the telehealth and group settings. However, there is room for refinement of mindful exercise as a smaller proportion of participants provided positive ratings and adherence was suboptimal (54%). This was likely related to duration of each session (∼2 ​h) with participants preferring shorter but more frequent sessions (Table 4). Similar low attendance (46.8%) with longer session duration (3 ​h) was observed in a study that provided MBSR and exercise online for 8 weeks [36]. Adherence was also negatively impacted by 4/21 participants who did not attend any sessions and were eventually lost to follow-up for reasons unrelated to the study. Among those who attended at least one session, the average attendance was 67% which is within the range observed for mindfulness programs [18,37], but lower than supervised, video-based, exercise programs for knee OA [38,39]. Further areas of refinement that we will consider for future studies include reinforcements for those who find mindfulness challenging, individualized exercise guidance, availability of intervention session recordings for home practice, and accommodation for peer interaction within the group. These suggestions are aligned with intervention design in prior studies with high attendance [38,39].

Our decentralized approach was feasible for recruitment (≥40 enrolled over 6-months). Of the people who completed online screenings, <10% were enrolled in the study. These rates are similar to what has been observed for decentralized studies [40]. For future studies, we will modify the digital advertisements to facilitate improvements such that individuals can self-screen as ineligible prior to completing the online screening form [40]. While our digital recruitment strategy was successful at reaching individuals across US including those from rural areas, it failed to reach individuals from minoritized racial groups. Adequate racial and ethnic diversity is essential in clinical research for a robust demographic representation that can enhance generalizability of findings. Interestingly, lack of racial and educational diversity has been noted in decentralized research designs, due to the technological divide and lack of access to digital technologies [41]. Therefore to improve diversity in future trials, we will supplement digital recruitment with strategies such as direct recruitment from hospitals, community-based groups, and use data-driven techniques. Our decentralized approach yielded excellent retention with >80% of participants providing outcomes at both 8-week and 14-week timepoints. The participant withdrawal rate in our study (12.5%) was lower than the median participant withdrawal rate (27%) observed for digital pain management interventions [42].

Our study was not designed to determine the efficacy of mindful exercise. Hence, our findings related to PROs are preliminary and should be interpreted as such. We observed numerically larger improvements in several PROs for mindful exercise group vs exercise group with some meeting criteria for minimal important change. These include knee pain during daily activities (NRS-na), pain interference (PEG), and quality of life (KOOS QOL). Although we did not see large improvements in fear avoidance, these results provide preliminary support for the premise of our mindful exercise intervention. Importantly, we observed numerically larger and clinically meaningful improvements in the mindful exercise group at week-14 for several KOOS subscales, NRS-na, and PEG. Similar findings were reported by Bagheri et al., where women with patellofemoral pain who received MBSR and exercise showed greater improvements in pain at 18-week and 2-months follow-up compared to improvements at 9-weeks [43]. Two other studies reported greater improvements in pain with the combination of mindfulness and exercise compared to controls (treatment as usual, audiobook control) in individuals with fibromyalgia and chronic low back pain [44,45]. However, to our knowledge, no prior studies have integrated mindfulness with exercise and typically provided mindfulness and exercise as separate interventions.

The goal of this study was to determine the feasibility of the mindful exercise intervention and our decentralized study design. Our study has provided several important findings that we can use to further iterate the mindful exercise intervention. While several of these modifications have been mentioned earlier, these could include shorter and more frequent intervention sessions, additional practice with mindfulness prior to initiating combined mindfulness and exercise, individual assessment and exercise prescription, strategies to further support intervention adherence, alternate recruitment strategies to increase racial diversity, and refinement of advertising to improve yields. These modifications would then need to be evaluated using another feasibility study (i.e., NIH Stage 1B) prior to efficacy studies. Our feasibility study was not intended to provide data to guide the choice of primary or secondary endpoints or the sample size for the future efficacy study. The guidelines for outcomes in clinical trials for knee OA will be used to define the primary and secondary endpoints in the future efficacy study. The sample size will be determined using published data on minimal important between-group difference in the primary endpoint(s).

There are limitations to consider while interpreting the findings of our study. First, all outcomes are participant-reported which can introduce bias. Therefore, we will include objective measures of physical performance and physical activity in our future trial. Second, our cohort lacked racial diversity so findings may not be generalizable to diverse populations. Finally, we did not include a mindfulness-only group so it is not clear if mindful exercise may be more beneficial than mindfulness alone. However, prior studies of mindfulness alone show smaller effect sizes as compared to effect sizes from exercise only studies [46,47]. Therefore, we chose to compare mindful exercise with exercise alone.

In conclusion, a supervised, telehealth, group-based mindful exercise intervention, developed by integrating mindfulness into strengthening exercises, is safe and satisfactory for people with knee OA. Our decentralized approach yielded excellent recruitment and retention but suboptimal adherence and racial diversity. The findings from this study will guide refinements to the mindful exercise intervention and study design to facilitate greater adherence and racial diversity.

## Author contributions

Conception and design of the study, or acquisition of data, or analysis and interpretation of data: All authors.

Drafting the article or revising it critically for important intellectual content: Shah, Morone, Ellis, Cohn, LaValley, Kumar.

Final approval of the version to be submitted: All authors.

## Role of the funding source

Investigators were supported by Boston University Sargent College Student Research Grant, Rheumatology Research Foundation Medical and Graduate Student Preceptorship Award, and National Institute of Health K01AR069720.

## Declaration of competing interest

None.
